# What matters to you? Public and patient involvement in the design stage of research

**DOI:** 10.1186/s40900-024-00610-1

**Published:** 2024-09-30

**Authors:** Amanda Hensman-Crook, Lois Farquharson, Juliette Truman, Catherine Angell

**Affiliations:** Bournemouth University, Bournemouth, UK

**Keywords:** Public and Patient involvement, Design phase of research, Influence, Impact, Accountability, Ethics

## Abstract

**Background:**

Public and patient involvement is critical to ensure that research is relevant and addresses what matters most to the person through co-production. Involvement at the design stage where ideas for research are developed prior to formal ethical approval, can positively influence the direction of research design, methods, and outcomes. Although ethical approval is not required at this stage, being ethically conscious is imperative to prevent unwarranted unethical practices. To ensure this, the public and patient intervention at the design stage of a doctoral research project was benchmarked against Pandya-Woods 10 ethically conscious standards and the INVOLVE values and principles framework. Ethical approval was also gained for publication.

**Main body:**

Patient and public involvement was undertaken with two diverse patient and public groups as an agenda item in their regular Teams meeting. Thoughts on the research project, the timeline, what matters most to the individuals in the group with regarding the design and outcomes from the research, the best method for data collection for public research, and next steps were discussed.

**Conclusion:**

Public and patient involvement had a positive influence on the design and outcomes of a doctoral research proposal and held the researcher accountable for impact of the research on the public. Positive changes to the research from working with public and patients exploring ‘what matters to you’ included: An ontological change in the way that the research was conducted, identification of some main themes to run as a thread throughout the research, development of content for an international scoping review, identification of the best method for data collection for patient research, and accountability of the researcher to write a plain English summary at the beginning of each thesis chapter, and a summary report at the end for dissemination.

**Supplementary Information:**

The online version contains supplementary material available at 10.1186/s40900-024-00610-1.

## Abstract (plain language summary)

Public and patient involvement (PPI) is encouraged at every stage of research to make sure that research is relevant, and addresses what matters most to the person by working together as equal partners . This commentary reflects on how PPI at the beginning of a research idea can positively influence the design, methods, and outcomes of the research. This stage of research is known as the ‘design phase’. It is important that although ethical approval is not needed for this phase, that the researcher acts in an ethically conscious way. The doctoral research ‘What factors and influences demonstrate quality and impact of the Southeast Consultant development Programme?’ commented on in this paper, has been mapped against some standards and has gained ethical approval for publication.

Two public and patient groups (PPG) were approached to be involved in the design phase of this research as an agenda item in their regular meeting. The PPG lead sent an involvement information sheet and consent form prior to the meeting to aid decision making whether to attend or not. Those involved could leave at any point and all discussion was anonymised.

The PPI positively influenced the research in the following ways:

- A change in the way that the research was conducted.
- Identification of main themes to be addressed based on ‘what matters to me’.
- Development of content for an international scoping review.
- The best method for patient research.
- Accountability held of the researcher to write a plain English summary at the beginning of each thesis chapter and a summary report at the end.

**Supplementary Information:**

The online version contains supplementary material available at 10.1186/s40900-024-00610-1.

## Background

Researchers, including doctoral researchers [9], are encouraged to involve patients and the public throughout all stages of research including the design phase, the focus of this commentary, where ideas for research are developed prior to formal ethical approval [19]. Contemporary literature identifies that PPI improves the quality and impact of health research [8, 9, 21, 29, 33] and provides the necessary assurance of accountability and transparency to the general public [18, 30]. PPI at all stages of health and social care research is of great importance to ensure that research is relevant and addresses what matters most to the service user [5, 14, 28] through co-production [17]. The importance of PPI and the researcher working together at this early stage to seek people’s input to inform and influence decisions about how research is designed, undertaken, and disseminated is well documented [12, 23–25, 27]. INVOLVE describes Patient and public involvement (PPI) as research carried out ‘with’ or ‘by’ people rather than ‘to’, ‘for’ or ‘about’ them [12] and should not be confused with qualitative research where a research question is answered using a specified method [10, 13].

It was decided to use both the Pandya-Woods conceptual Framework [19] (Table 1) and the INVOLVE ‘Public involvement in research: values and principles framework’ [12] (Table 1), to inform the design phase of the doctoral research commented on in the main text of this paper to add quality [5, 14, 28] and to prevent tokenism [31]. Further, for transparency, rigor and accountability, ethical approval was gained from the Bournemouth Universities ethics committee to enable publication.

**Table 1:** Mapping to INVOLVE [12] and Pandya-WoodI at the design stage of research [19]

INVOLVE Public involvement in research: values and principles	Evidence of mapping to the INVOLVE and Pandya Wood statements	Pandya- Wood areas of concern at the design phase of research statements
**Respect** Researchers, research organisations and the public respect one another’s roles and perspectives	PPI groups were sent details of the purpose of PPI involvement, their role, and the researcher’s role to be held as an agenda item on their next meeting 2 weeks before the meeting. This allowed time to read and to ask questions before deciding if they would like to contribute Although there is no requirement to go through ethical approval for Public and Patient involvement this was sort to ensure accountability through university ethics PPI will be acknowledged for their contributions in this paper and will be in the subsequent PhD thesis Public and patients who were involved were provided clarity of both the researcher’s and their roles prior to deciding if they wished to contribute and had the opportunity to opt out at any point should they wish to do so without explanation Sensitivity was always adhered to throughout as aware that several contributors in the group have lived/carer experience of the topic under investigation. Care was taken to avoid any potentially emotionally upsetting or sensitive subject matter The PPI groups will be acknowledged in any published paper relating this design stage of the research as a group and will have the publication disseminated to them via their PPI group lead PPI groups will be acknowledged in the doctoral thesis and a plain English summary at the beginning of each chapter will be written as requested and summarised in a combined report on completion of the thesis	1 Allocating sufficient time for public involvement 2 Avoiding Tokenism 3. Registering of research design stage public involvement work early with NHS Research and Development (R&D) Trust Office 4. communicating clearly from the outset 5. Entitling public contributors to stop their involvement for any unstated reason (s) 6. Operating ‘fairness of opportunity’ 7. Differentiating between public activities and qualitative research methods 8. Working sensitively 9. Being conscious of confidentiality 10. Valuing, acknowledging, and rewarding public involvement
**Support** Researchers, research organisations and the public have access to practical and organisational support to involve and be involved	The researcher had supervisory support for engagement with PPI at the design phase of research and was signposted to relevant papers and websites PPI were given the opportunity for support prior to, throughout and after the meeting(s) from the researcher PPI were supported to stop their involvement at any point should they wish to. The groups had access to contact information of the researcher Sensitivity was always adhered to throughout as aware that several contributors in the group have lived/carer experience of the topic under investigation. Care was taken to avoid any potentially emotionally upsetting or sensitive subject matter A mechanism for counselling via the PPI group lead was put in place should any arise prior to undertaking the agenda item consultation	1 Allocating sufficient time for public involvement 4. Communicating clearly from the outset 5. Entitling public contributors to stop their involvement for any unstated reason (s) 8. Working sensitively
**Transparency** Researchers, research organisations and the public are clear and open about the aims and scope of involvement in the research	A Teams meeting with the two PPG group leads acting as facilitators, were held prior to the scheduled consultation with the groups. This was an initial clarification role, expectations, and the level of involvement regarding the duration and type of contribution from the group, and to gain permission for them to distribute relevant paperwork to the group prior to the meeting Plain English language was used in an information sheet to ensure that there was no confusion between the PPI group involvement as contributors to inform the design of the PHD project prior to ethical approval for the research to be undertaken refined by their thoughts and comments The involvement with the PPI group was an agenda item in their regular meeting and was designed to encourage conversation around the topic, not as a research project It was agreed that anonymous summary bullet points could be collected of the main points raised that would subsequently direct the project design. These were agreed following the meeting as ‘minutes’ of the agenda item A written explanation of the above following the Teams meeting was sent to the PPI groups by the PPI group lead prior to the meeting for the PPI group to read to aid understanding, to ask any questions prior to the meeting and to help with informed decision making to decide if the wished to attend the consultation. Other papers were also sent including a PPI information sheet regarding the project, a consent form, Ghant Chart, and proposed project plan A PPI information sheet was sent to all involved and a consent form which clearly state that they can opt out at any stage with no explanation Approval was sort for this paper for transparency and accountability Clarity was given that there would be no financial reward to being involved. Involvement was an agenda item in a regular unfunded PPI meeting	2 Avoiding Tokenism 3. Registering of research design stage public involvement work early with NHS Research and Development (R&D) Trust Office 4. communicating clearly from the outset 7. Differentiating between public involvement activities and qualitative research methods
**Responsiveness** Researchers and research organisations actively respond to the input of public members involved in research	A 2-week window was given following the distribution of information to allow the public members to contact the researcher or group lead to answer any questions Although there was a structure for the agenda item, throughout the meeting(s) there was flexibility to voice opinions at any time point to allow points to be clarified or expanded, and to enable everybody to have a voice in the room From the initial involvement at the design phase of the project, it was requested from both groups that they would have an opportunity to be involved in reviewing questions in each research package prior to submission to ethics A request was made that a plain English summary is added to each chapter of the PHD thesis and pulled together as a paper at the end for them to see the outcomes of the research and how their input was incorporated into the objectives and outcomes Contact for the researcher was given to the group	1 Allocating sufficient time for public involvement 2 Avoiding Tokenism 8. Working sensitively 7. Differentiating between public activities and qualitative research methods 10. Valuing, acknowledging, and rewarding public involvement
**Fairness of opportunity** Researchers and research organisations ensure that public involvement in research is open to individuals and communities without discrimination	The PPI groups were from diverse backgrounds Two PPI groups were involved, one a PPI group who have had prior involvement in PPI research and another group who have had no prior involvement in PPI research	2 Avoiding Tokenism 8. Working sensitively 6. Operating ‘fairness of opportunity’ 10. Valuing, acknowledging, and rewarding public involvement
**Accountability** Researchers, research organisations and the public are accountable are accountable for their involvement in research and to people affected by the research	Although ethical approval is not required at the design (ideas) phase of research, it was decided that ethical approval would be sought for transparency and accountability A clear explanation of the difference between ‘involvement’ and research was given Confidentiality was adhered to and addressed in the PPI information sheet and consent form There was no recording undertaken and any quotes were not written directly, rather as a summarised bullet point to protect anonymity Keeping PPI groups informed prior to the meeting(s) and after the meeting(s) PPI groups were invited to take part in reviewing the questions for the research packages prior to submission for ethical approval The researcher is being held directly accountable by the PPI groups for the publication of a paper of PPI input at the design phase of research and to produce chapter summaries at the beginning of each chapter in the PHD thesis and summary report of all chapters on completion of the thesis A mechanism for counselling via the PPI group lead was put in place should any arise prior to undertaking the agenda item involvement	2 Avoiding Tokenism 3. Registering of research design stage public 5. Entitling public contributors to stop their involvement for any unstated reason (s) 7. Differentiating between public activities and qualitative research methods 9. Being conscious of confidentiality

As the doctoral research to be undertaken is health and social science in nature, the public and patients are end users, so their views are critical to ensure what matters most to them is central. It was therefore important that those involved were representative of a diverse population and included diversity within groups who experience of health and social science and those with no experience were included to ensure depth and breadth.

The aim of this paper is to explore the impact of public and patient involvement in the co-production of a doctoral research project in the design phase of research conducted in an ethically conscious manner.

## Main text

### Doctoral project

The doctoral project ‘What factors and influences demonstrate quality and impact of the Southeast Consultant development Programme?’ is the focus of comment in this paper in relation to the influence of PPI on research design, methods, and outcomes in the early ideas stage of research. This project is now underway following PPI involvement and will be completed in September 2025.

**Fig. 1:**
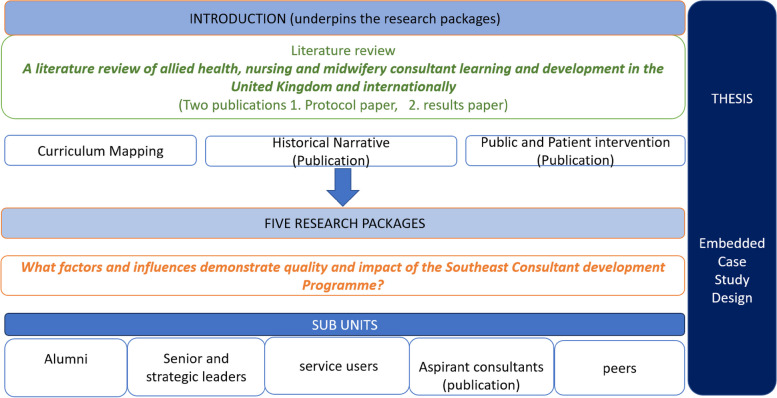
Doctoral Project outline

This project, seen in Fig. 1, is an embedded single case study design [3] that focuses on multiple parts (the ‘sub-units’ and underpinning introduction) of a single case (the Southeast Consultant Development) chosen to allow a range of methods including qualitative, quantitative, archival, and mixed methods to investigate the case [26, 32]. The original ontological position was phenomenological [6, 20, 22] to explore the doctoral projects aims and objectives from the lens of the lived experience of the individual participants [1].

### Overall aim and objectives of the doctoral research

#### Aim

To investigate the evolution of the Southeast consultant learning and development programme in the context of policy and strategic drivers, and to investigate the current educational programme to determine if it meets expectations of quality and impact from multiple perspectives.

### Objectives:

- To engage with public and patients at the design phase of the research to inform the design, methods and direction of the project.
- To undertake an international scoping review to explore consultant level practice, the learning and development of, accreditation, regulation, and revalidation from a global perspective: *‘An international scoping review exploring the definitions of, and the learning and development pathways leading to expert practice across nursing, midwifery, and the allied health professions.’*
- Historical narrative to explore the evolution of the Southeast consultant learning and development programme in the context of political and strategic milestones: *‘Historical narrative of the evolution of the southeast consultant development programme in the context of political and strategic milestones.’*
- Curriculum mapping of the Southeast Consultant programme to the multi-professional consultant-level practice capability and impact framework [11] to identify any gaps or redundancies.
- Exploration of the Southeast consultant learning and development pathway from different perspectives in relation to factors affecting quality and impact
  Sub-unit 1: Aspirant Consultants
  Sub-unit 2: Patient
  Sub-unit 3: Senior and Strategic leadership
  Sub-unit 4: Programme Alumni
  Sub-unit 5: Peers

### PPI Process

Two separate existing diverse (range of ages, genders, ethnicity, and geographical location) Public and Patient groups (PPG) were approached that had a combination of healthcare professionals and others outside the profession; one a PPG experienced in involvement with Health Science research (*n* = 6) who regularly had PPI on their meeting agenda, and a second group with no health science research experience (*n* = 5) who had never had PPI on their meeting agenda. Evidence shows that training and experience in health research of contributors of PPI has a positive effect in addressing potential imbalances such as misunderstanding of medical jargon or of research processes supporting conversation [21]. Equally, the value of having no health research experience to bring purely experiential knowledge to the discussion by eliminating any form of ‘professionalism’ is also recognised to inform projects [31]. It was therefore decided that both groups would be included to capture both schools of thought for diversity of opinion.

The PPI took place in one group session as the last agenda item within their scheduled PPG Teams meetings to enable those who did not want to be involved to leave. Prior to the meeting, the PPG leads as facilitators, were contacted to distribute an involvement information sheet outlining the project, the purpose of the agenda item and a consent form. Those who agreed to be involved, could opt out at any stage with no explanation. The agenda item was not recorded. Points of discussion were summarised anonymously in written bullet points during the meeting, checked by the PhD supervisory team, and sent out via the PPG leads for distribution to the PPI group following the meeting as minutes for comments of accuracy.

### Content

The PPI was conducted by the lead author for one hour as the last agenda item to enable those who wished to leave to do so, and to ensure there was time to complete the points of discussion. Points of discussion were divided into:

- Thoughts on the research project (including ontological position) and the proposed timeline.
- Discussion of what matters most to the individuals in the group with regards to outcomes from the research.
- Discussion regarding be the best method for data collection for the public and patient arm of the research.
- to discuss next steps

### Impact and change from PPI group discussion

- Thoughts on the research project as a whole and the proposed timeline.

There was a consensus of approval of the embedded single case study design of the project and the proposed timeline. It was felt that the embedded single case study design enabled flexibility in methodology to provide a comprehensive underpinning narrative for the project, and to explore the sub-groups individually, across the sub-groups, and in relation to the case to meet the aim and objectives of the project.

Both groups questioned phenomenology as the ontological approach as it would limit the research to qualitative methods. It was widely felt that a realist approach would be a better fit for the project as supports the multi-methodology of a case study design, and is commonly used in social sciences and education [2, 4, 15, 16] to find truth through competent enquiry [7].

- Discussion of what matters most to the group with regards to the design and outcomes from the research.

‘What matters most’ was broadly the same across both groups and can be summarised into three main subject areas: PPI, impact (a. of the environment/culture, b. in relation to evidence) and academic learning and development content (a. in relation to embedding personalised care, b. in relating to academic/flexible learning). Expanded summaries of these subject areas, and the influence on the research can be seen in Table 1 below (Table 2).

**Table 2:** Thoughts and impact of discussion of desired outcomes from the project

Summarised discussion theme	Summarised conversation in nominal sentences from the PPG groups	Influence on the research
**PPI needs to be embedded at all levels of education**	Learning at all levels should embed PPI embed into it Communication / feedback skills should be incorporated e.g., a practical session with PPI (supported by training) demonstrating different emotions such as anger or fear to learn how to be in those situations – how somebody feels is really important and individual depending on the person and the situation. This seems to be lacking in education as not translated in the real world Need to incorporate how to build trust. Need experience in practice to learn skills to build trust. Maybe incorporate PPI into the role play exercises above? Would like to know at what level PPI is genuinely embedded into the consultant programme in HEIs – in research at all stages and in practice Have PPI to write research summaries in plain English for public and patients to understand Genuine co-production and PPI and patients important, not just a tick box. This should be built in throughout the career pathway and should be transparent Having PPI throughout all learning and development is critical Co-production with PPI to run throughout all development undergraduate to consultant – wrapped around and integral to it PPI at interviews for educational programmes and consultant recruitment to prevent bias and improve quality of selection	PPI will be embedded as a thread running throughout the research as one of the factors and influences affecting the quality and impact of learning and development programmes for non -medically trained consultants PPI will be used as part of reference groups for question design for every part of the research PPI will help inform the plain English summaries at the beginning of each thesis chapter
**Impact**	**a) Environment/culture** There is a silo mentality and lack of parity in the NHS, non-medically trained consultants need to have parity with medical consultants An aspiration should be to have a genuinely holistic approach to patient care where clinicians work together to ensure the best outcome rather than working in silos on medical and not medical consultants Need to consider what impact the environment has on a non-medically trained consultant to them being able to apply their learning into practice from a managers and peer perspective **b) Evidence** Clear recommendations from research backed up by evidence of change Evidence of impact from implementation of research into practice Plain English paragraph from a patient’s perspective of how contact with a non-medically trained consultant affects them Impact is important – the translation of knowledge into practice for the research and professionals – needs to be measured Findings need to be presented in a plain English paragraph so that the public can be informed and can comment on	The impact of the working environment and the opinions of peers will be included as an embedded thread throughout the research as one of the factors and influences affecting the quality and impact of learning and development programmes for non -medically trained consultants Recommendations from the research will be produced in plain English of areas where, and how, a positive impact for the public and patients can be made in relation to the training of and the implementation of non-medically trained consultants
**Academic/ Learning and development content**	**a) Embedding personalised care** Promotion and elevation of different specialisms feels important to deliver personalised care rather than a specific profession. If delivering personalised care in this context, it would also help move away from the ‘doctor is King’ mentality There needs to be shared decision making and personalised care embedded into a learning and development programme as consultants are responsible to translate that learning into practice. Consultants often too far removed from the general public because they don’t have this skillset Needs skills to ensure that the public and patient voice is heard as an equal in every consultation. E.g. Ask questions such as: “What do you want?”, “What do you think we should do?”, “What are your aspirations for your life?” and “Do you have targets for the next year?” The ability to apply knowledge into practice including communication skills with the person as an equal in shared decision making with ‘what matters to (the person) them’ in the centre at every stage of care. Need to be able to deal with emotion. It is how a professional makes you feel that is important What Matters To You: All clinicians and consultants should set the example, should be asking patients what matters to them at that point in time. Using this as to then tailor the approach to treatment and care in a way that is personalised to the individual **b) Academic v flexible learning** A rigid standardised academic programme would create inequality, needs to be flexible between academic and practical learning ‘The use of words or equivalent’ is important. People with a certain qualification can pass the requirements for the qualification but may not be able to do the job Life experience is important to be able to do the role not just the ability to pass exams Accreditation at an agreed level will ensure capability and impact but it needs to be an individual flexible development programme to ensure that any gaps are filled that may be the ability to personalise care, c Looking at facilitators and challenges to the outcomes of patients There needs to be teaching and assessment of how to communicate with people- can they apply knowledge into practice for example Ongoing CPD is really important. It is not good enough to tick the box of a qualification and never be tested again. Need to be re-evaluated e.g. on a 3 yearly basis with a practical element to the re-evaluation with PPI involvement either as an observer or participant It would be helpful if consultants spoke and wrote in English. Maybe a part of recruitment and the training should include this as this has a direct impact on patient care?	Personalised care will be included as an embedded thread throughout the research as one of the factors and influences affecting the quality and impact of learning and development programmes for non -medically trained consultants The scoping review will be international to ensure breadth and depth to explore the different learning and development models of non-medically trained consultants The use of the word ‘equivalent’ and ‘CPD’ will be used in the search terms of the scoping review Accreditation and revalidation will be included in the scoping review ‘Experience’ will be included as part of the discussion in the scoping review

### Discussion regarding be the best design for data collection for the public and patient part of the research

It was broadly agreed that a digital questionnaire with open and closed questions would be the best design for data collection for the public and patient part of the research. Having open qualitative questions to support quantitative data would add depth to the research. This method was given with the caveat that it would be made accessible by ensuring that in the information sheet it was clear that they can have help from a trusted person if unable to access or understand digital technology.

### To discuss next steps

Both groups requested that each chapter of the doctoral thesis has a plain English summary at the beginning of the chapter, with one group adding that the summaries are combined into a final plain English report of the thesis for dissemination. It was felt that for the new knowledge generated by the research to be useful and impactful, it needed to be accessible for the public.to read, understand, and have the opportunity to comment.

It was further requested by both groups for ongoing PPI to be part of the review process for the content of draft research questions prior to submission to ethics. This would ensure accountability and prevent PPI tokenism.

Engagement with PPI as outlined above has since led to many changes to the doctoral research moving forward that include: The ontological stance of the research, identification of some main themes to run as a thread throughout the research, development of content for an international scoping review, identification of the best method for data collection for patient research, accountability of the researcher to write a plain English summary at the beginning of each thesis chapter, and a summary report at the end for dissemination for public review.

## Conclusions

Public and patient involvement has a positive influence on the design and outcomes of a doctoral research proposal and held the researcher accountable for the impacts of the research on the population when conducted in an ethically conscious way. Co production with public and patients in the design phase led to significant changes that would not have been considered without exploring the project from the paradigm of ‘what matters to you’ in this early stage. Changes included: The ontological stance of the research, identification of some main themes to run as a thread throughout the research, development of content for an international scoping review, identification of the best method for data collection for patient research, accountability of the researcher to write a plain English summary at the beginning of each thesis chapter, and a summary report at the end for dissemination for public review.

Although two different groups of PPG were utilised; one with experience of PPI in health and social science research and one with no experience, there was very little difference in the subsequent themes that emerged, only in the language used to express it.

Care needs to be taken whilst working with the public and patients in the design phase to ensure that PPI is conducted in an ethically conscious manner. The use of ethical benchmarking against standards such as Pandya-Woods conceptual framework and the INVOLVE values and principles framework helps to mitigate unwarranted unethical practice, and prevent PPI tokenism, by raising the consciousness of the researcher whilst engaging with public and patients. Further, this helps to ensure that PPI in the design phase is not confused with qualitative research which requires formal ethical approval.

Working with public and patients in the design stage of research is a rewarding experience that enhances the quality and impact of the research attuned to what matters most to the person.

## Supplementary Information

Additional file 1: Table x GRIPP Short form [28].

## Data Availability

No datasets were generated or analysed during the current study.

## Abbreviations

PPI: Patient and public involvement
PPG: Patient and public group
PhD: Doctor of philosophy
