# Supplementary material for: What matters to you? Public and patient involvement in the design stage of research
Source: Res Involv Engagem. 2024 Sep 30;10:100. doi: 10.1186/s40900-024-00610-1 (PMC11440653; doi:10.1186/s40900-024-00610-1)
Supplement: Supplementary file 1 — Additional file 1: Table x GRIPP Short form [28]. [file 40900_2024_610_MOESM1_ESM.docx]

| Section and topic | Item | Reported on page number |
| --- | --- | --- |
| Aim | Report the aim of PPI in the study | 5 |
| Method | Provide a clear description of the methods used for PPI in the study | 13-14 |
| Study results | Outcomes—Report the results of PPI in the study, including both positive and negative outcomes | 14-20 |
| Discussion and conclusions | Outcomes—Comment on the extent to which PPI influenced the study overall. Describe positive and negative effects | 14-21 |
| Reflections/Critical appraisal | Comment critically on the study, reflecting on the things that went well and those that did not, so others can learn from this experience | 20-21 |

Table x GRIPP Short form [28]
